# Investigating the impact of environmental feedback on the optional prisoner’s dilemma for insights into cyclic dominance and evolution of cooperation

**DOI:** 10.1098/rsos.240717

**Published:** 2024-10-23

**Authors:** Md. Fahimur Rahman Shuvo, K. M. Ariful Kabir

**Affiliations:** ^1^ Department of Mathematics, Bangladesh University of Engineering and Technology, Dhaka 1000, Bangladesh

**Keywords:** evolutionary game theory, cyclic dominance, optional prisoner’s dilemma, rock–paper–scissors, environmental feedback

## Abstract

This study incorporates environmental feedback into the optional prisoner’s dilemma and rock–paper–scissors games to examine the mutual influence of eco-evolutionary outcomes and strategy dynamics. A novel game-theoretic model is developed that integrates the optional prisoner’s dilemma and rock–paper–scissors games by incorporating an environmental state variable. By adjusting feedback parameters, chaos, oscillations and coexistence are observed that surpass the usual outcomes of social dilemmas when the environment transitions between depleted and replenished states. Defection is no longer advantageous in evolution; cooperation, abstention and cyclic dominance arise. The observed transitions align with natural economics, ecology and sociology phenomena. The inclusion of abstention options and environmental feedback has a significant impact on collective outcomes when compared with conventional games. This has important implications for studying adaptation and decision-making in situations with ecological constraints.

## 1. Introduction

This study presents the concept of cyclic dominance in a pairwise game framework obtained from the convex combination of the optional prisoner’s dilemma (OPD) game and the rock–paper–scissors (RPS) game through environmental feedback (EF). The combination creates an eco-evolutionary two-player, three-strategy game called the Pairwise Loner Game (PLG). The methodology utilized in our study involves the implementation of universal dilemma scaling within a conventional prisoner’s dilemma (PD) framework, supplemented by introducing a new strategy called the loner [1]. This study aims to study the effects of dilemma, abstinence, EF and cyclic dominance occurring simultaneously.

Evolutionary game theory examines how strategic interactions among rational decision-makers evolve within a population, considering the incentives provided through strategy-dependent pay-offs [2,3]. The popularity of a particular strategy is determined by the dynamics of the system and the incentives provided to individuals for selecting that strategy. This can result in myopic behaviours, where the preferred individual option is not necessarily the best choice for the overall population. The situation described pertains to the widely recognized PD, where the most advantageous course of action is to defect [4]. The PD game has been widely employed to examine the development of cooperation in social dilemma games since it effectively illustrates the clash between individual and collective interests [5]. Cooperation can still arise by allowing players to penalize defectors, incentivize cooperators or choose not to participate (loner strategy) [6–11]. One of the most significant types of third strategy is the optional or voluntary participation in social dilemmas and public goods games, where loners influence the evolutionary dynamics along with traditional cooperators and defectors [9,11–13]. This formulation incorporates the dynamics observed in various real-life decision-making scenarios, such as the spread of opinions during an election campaign and the competition between two dominant firms in the marketing industry [14,15]. The inclusion of the loner strategy enables the simultaneous presence of cooperators, defectors and loners through a pattern of cyclic dominance in well-mixed populations [16–18]. Cooperators receive higher pay-off than loners, loners are immune to harm from defectors and defectors naturally dominate cooperators, thus maintaining cooperation while strategy frequencies oscillate. This pattern is similar to the dynamics observed in the RPS game, a common framework to study cyclic dominance [19]. Cyclic dominance can be observed in various situations beyond the context of the OPD including the public goods game with linked punishment and reward [20,21] and the study of biodiversity and competition among microbial communities [22–24]. The RPS game, known for its depiction as the quintessential example of cyclic dominance, has been applied in diverse domains, such as lizard reproductive strategies [25], bacterial antibiotic production [24], mechanical engineering and geometric configurations [26].

Nevertheless, irrespective of the mathematical model employed, the typical game scenario entails predetermined rewards linked to each strategy. This limitation fails to account for the influence of the environment on game dynamics and, consequently, the frequency of strategy within the population. The research conducted by Weitz *et al*. [27] has generated significant interest due to its novel approach to modeling the interdependence between pay-offs and the environment. This approach considers the impact of enhancement and degradation effects on an environmental resource. Such interactions are prevalent across various fields, such as sociology, economics and animal behaviour [28–31]. An instance of this phenomenon in human systems is the individuals’ choice to either vaccinate or abstain from vaccination, leading to the occurrence of preventable infectious diseases in children [32–36]. Consequently, there is a growing motivation to vaccinate. The concept of game–environment feedback, known as eco-evolutionary game theory, has gained significant attention in the control community. In this context, developing effective control policies is essential for achieving the desired system behaviour [37,38]. The study of honeybees' collective decision-making process has recently involved analysing the optimal control problem [39]. In this context, the parameters of the mean-field game model serve as implicit EF.

Su *et al*. [40] have explored a new approach by examining how the environment and its changes affect game transitions. This involves a framework where players switch from one game and its corresponding pay-off matrix to another based on the environmental resource. Jia *et al*. [41] examined a model of the RPS game that incorporates EF where a replenished environment offers an additional reward for the confrontation games.

These studies utilizing convex combination of different games’ pay-off structures demonstrate the complex interactions that can occur when games are designed to rely on and impact a dynamic environment. Connecting separate games through EF offers a seamless method for representing strategic shifts and the simultaneous development of behaviours in response to ecological circumstances. An illustrative example of the framework can be seen in the context of environmental conservation efforts, such as a community that relies on water or fisheries. In situations where resources are limited, individuals may be tempted to exploit these resources for their personal gain, leading to a phenomenon called the tragedy of the commons [16,42]. In this situation, people are confronted with a dilemma where they have to choose between collaborating by practising sustainable resource management or acting against the common interest by excessively exploiting resources for immediate personal benefit. In contrast, in situations where there is an ample supply of resources, various stakeholders, including environmental agencies, local communities and businesses, may adopt a range of strategies to make use of those resources. These strategies may engage in cyclic competition, akin to the dynamics of the RPS game [24,43].

This study aims to create a game theoretic model that connects OPD frameworks to RPS frameworks in an eco-evolutionary game. As previously mentioned, the OPD framework demonstrates cyclic dominance similar to RPS games. Therefore, a comprehensive analysis is necessary when integrating the OPD and RPS frameworks. An ecological and evolutionary relationship between the two frameworks is formed through EF, which provides a new way to investigate the interconnected games. Several previous studies have employed environmental conditions to integrate two game frameworks [13,40,41,44–46]. However, only a limited number of studies have specifically investigated the relationship between the OPD and RPS frameworks using environmental conditions. The majority of these studies employ two strategies. Insufficient research has been conducted on games with three strategies. This study also incorporates the concept of environmental tipping points [27,47] to facilitate the transition from a challenging situation with low environmental conditions to a more favourable situation with high environmental conditions. It is worth noting that this aspect has not been extensively explored in existing literature. It can be inferred that a favourable environmental condition fosters competition, as seen in games like RPS, whereas an unfavourable environmental condition gives rise to a dilemma. In order to fully understand the oscillatory tragedy of the commons, it is crucial to conduct comparative studies on three strategies, particularly those that involve cyclic dominance. This is because two strategy games have already provided evidence of this phenomenon. It is imperative to address this deficiency in order to fully understand the process of decision-making in intricate systems and develop strategies to mitigate adverse outcomes in comparable game situations.

The study introduces an interesting framework that intricately links EF with the emergence of dilemmas, loner behaviours and cyclic dominance phenomena. This framework represents a significant advancement in understanding the dynamics of strategic interactions within cyclic triad scenarios. By leveraging evolutionarily stable strategies (ESSs), evaluating the influence of various parameters, and employing numerical solutions, the framework sets forth precise criteria for the evolution and extinction of strategy. Through this comprehensive approach, the study offers insights into the intricate interplay between EF and strategic behaviour, shedding light on complex ecological systems’ dynamics. The study begins by introducing the game-theoretic model, followed by a detailed explanation of the game’s evolutionary dynamics and the resulting equilibria in the game. The concept of ESS [38,48,49] and the replicator dynamics [27,50] from evolutionary game theory are helpful for analysis. This research primarily aims to investigate the core dilemma’s impact on the game’s structure and dynamics. Additionally, the impact of various parameter configurations on the game’s dynamics is comprehensively analysed.

## 2. Model

Consider a game played by two participants, wherein each participant is presented with the option to either cooperate or defect. When both participants opt for cooperation, they are both given a reward represented as R in recognition of their cooperative conduct. In contrast, if both players choose to defect, they will both experience a punishment P. In scenarios characterized by one player opting for defection while the other player chooses cooperation, the defector is rewarded with a pay-off T, referred to as Temptation, while the cooperator is subjected to a pay-off S known as the Sucker’s pay-off. The aforementioned fundamental framework can be concisely depicted using the subsequent pay-off matrix,


(2.1) $$\left[\begin{array}{cc} R & S\\ T & P \end{array}\right].$$

By including the concept of universal dilemma strength [51], the pay-off matrix in equation (2.1) is adjusted to be


(2.2) $$\left[\begin{array}{cc} R & P-D_{r}\\ R+D_{g} & P \end{array}\right]$$

where $D_{g}=T-R$ and $D_{r}=P-S$ are defined as the gamble-intending dilemma (GID) and the risk-aversion dilemma (RAD), respectively [51]. The GID parameter $D_{g}$ measures how much players are inclined to exploit each other. Mathematically, $D_{g}>0$ represent the temptation of defecting surpassing the reward for mutual cooperation where players are motivated to take advantages of cooperators. Conversely, $D_{g}<0$ represent that mutual cooperation is better than temptation. The RAD parameter $D_{r}$ measures how much players should refrain from exploitation. $D_{r}>0$ represent that the punishment from defection is greater in magnitude than being exploited and receiving a sucker’s pay-off. Individuals tend to punish defectors when $D_{r}>0$ while they prefer to establish cooperation when $D_{r}<0$.

Naturally, $D_{g},D_{r}\in \left(-\infty ,\infty \right)$ although it raises a critical concern if R-P is significantly different from $D_{g}$ and $D_{r}$ [52]. The game dynamics are significantly influenced by the values of $D_{g}$ and $D_{r}$, while the proportion of cooperators is dependent on R-P. If the value of R-P is significantly greater than both $D_{g}$ and $D_{r}$, the resulting effect is akin to approaching the limits of T→R and P→S [51]. The outcome becomes unaffected by a player’s choice and is solely determined by the opponent’s proposal. To resolve this situation, one possible solution is to assign R=1 and P=0, which represent the scaled best reward and scaled worst punishment, respectively. Additionally, it is important to restrict the values $D_{g},D_{r}\in \left[-1,1\right]$. This enables proportional relationships among R-P, $D_{g}$ and $D_{r}$. Moreover, the game dynamics are now contingent upon the sign and relative magnitudes of the $D_{g}$ and $D_{r}$ parameters, rather than their absolute values.

We set R=1 and P=0 and include the strategy, wherein individuals can refrain from engaging in the game. If either player opts for non-participation, they are both awarded the loner’s pay-off q. As a result, the OPD game is characterized by the following pay-off matrix:


(2.3) $$A_{OPD}=\left[\begin{array}{ccc} 1 & -D_{r} & q\\ 1+D_{g} & 0 & q\\ q & q & q \end{array}\right].$$

In order for the game to become a traditional OPD game, it must meet the condition of $1+D_{g}>1>q>0>-D_{r}$, which means that $D_{g}>0$, $D_{r}>0$ and 0<q<1. However, our study entails to $D_{g},D_{r}\in \left[-1,1\right]$ and $q\in \left(0,1\right)$ for a more holistic analysis.

The RPS game is based on the essential idea that paper defeats rock, rock defeats scissors and scissors defeat paper. Individuals are provided with three distinct strategies, namely rock (strategy R), scissors (strategy S) and paper (strategy P). Within the framework of this particular game, the participant who emerges as the victor is granted a pay-off of +1, whereas the defeated player receives −1 as a loss. A draw occurs when both players choose the identical strategy, resulting in both parties obtaining a payment represented as ϵ [53]. Equation (2.4) presents the payout matrix for the RPS game:


(2.4) $$A_{RPS}=\left[\begin{array}{ccc} \epsilon & -1 & 1\\ 1 & \epsilon & -1\\ -1 & 1 & \epsilon \end{array}\right].$$

In conventional RPS games, the value of ϵ=0 is commonly considered. In this study, we aim to investigate the effects of varying values of ϵ on the outcomes of draw and confrontational games. The resultant pay-off matrix for the overall game is derived by combining the PLG and the RPS game using a convex combination that is impacted by the environmental state, represented by the variable $n\in \left[0,1\right]$. The resulting form of the matrix is as follows:


(2.5) $$A\left(n\right)=\left(1-n\right)A_{PLG}+nA_{RPS}=\left[\begin{array}{ccc} \left(1-n\right)+n\epsilon & -\left(1-n\right)D_{r}-n & \left(1-n\right)q+n\\ \left(1-n\right)\left(1+D_{g}\right)+n & n\epsilon & \left(1-n\right)q-n\\ \left(1-n\right)q-n & \left(1-n\right)q+n & \left(1-n\right)q+n\epsilon \end{array}\right].$$

When the environment is completely depleted (n=0), the game strongly resembles the PLG. On the other hand, under the scenario when the environment is completely replenished (n=1), the game demonstrates a resemblance to the RPS game. We consider that the fractions representing the percentage of individuals who cooperate, defect and remain loners are denoted as x, y and z, respectively, while satisfying the condition x+y+z=1. Equation (2.6) presents a formal mathematical model that captures the game’s dynamics as experienced by the players, expressed through a set of differential equations:


(2.6*a*) $$\frac{dx}{dt}=x\left(\pi_{x}-\pi \right),$$

(2.6*b*) $$\frac{dy}{dt}=y\left(\pi_{y}-\pi \right),$$

(2.6*c*) $$\frac{dz}{dt}=z\left(\pi_{z}-\pi \right).$$

The equations are formulated based on the fitnesses $\pi_{x}$, $\pi_{y}$ and $\pi_{z}$ associated with the cooperators, defectors and loners, respectively, as well as the average fitness π of the total population. These fitnesses are further expounded upon in equation (2.7):


(2.7*a*) $$\pi_{x}=\left[\left(1-n\right)+n\epsilon \right]x+\left[-\left(1-n\right)D_{r}-n\right]y+\left[\left(1-n\right)q+n\right]z,$$

(2.7*b*) $$\pi_{y}=\left[\left(1-n\right)\left(1+D_{g}\right)+n\right]x+n\epsilon y+\left[\left(1-n\right)q-n\right]z,$$

(2.7*c*) $$\pi_{z}=\left[\left(1-n\right)q-n\right]x+\left[\left(1-n\right)q+n\right]y+\left[\left(1-n\right)q+n\epsilon \right]z,$$

(2.7*d*) $$\pi =x\pi_{x}+y\pi_{y}+z\pi_{z}.$$

The relationship between the environment and individual behaviour is inherently intertwined. It can be argued that confrontational games between different population strategies significantly contribute to ecosystem recovery, whereas draw games impede the replenishment of resources [41]. Multiple strategies engaging in confrontation spares their advances towards environment, leading to an increase in the environmental resource pool. When a specific strategy becomes excessively prevalent, it consumes upon the environment having a negative impact on the environment. The underlying assumption of the model is that the environment assumes the role of a participant in the game, albeit without actively choosing a strategy. The environmental fitness is contingent upon the actions of the agents and creates a reciprocal relationship between the strategies of the agents and the condition of the environment. The present study postulates that the environment undergoes replenishment at a rate of $\theta_{1}$, whereas depletion occurs at a rate of $\theta_{2}$. The formulation of the environmental influence function is as follows:


(2.8*a*) $$f\left(x,y,z\right)=\theta_{1}\left(2xy+2yz+2zx\right)-\theta_{2}\left(x^{2}+y^{2}+z^{2}\right).$$

To simplify, the environment depletion rate $\theta_{2}=1$ is assumed to be constant and the environment replenishment rate is replaced with a relative replenishment rate $\theta =\theta_{1}/\theta_{2}$. According to this framework, θ≥1 always guarantees that the environment will be replenished, while θ=0 always guarantees environmental degradation. Thus, we restrict the study to $\theta \in \left(0,1\right)$ in order to observe the inherent uncertainty within this range. This range is optimal for studying the interactions between strategies based on different combinations of parameters and the feedback from the environment. Thus, we have:


(2.8*b*) $$f\left(x,y,z\right)=\theta \left(2xy+2yz+2zx\right)-\left(x^{2}+y^{2}+z^{2}\right).$$

Equation (2.8*b*)’s environmental influence function does not differentiate between the impacts of cooperators, defectors and loners on environmental changes. This formulation is supported by examining a practical resource allocation situation in a community. For example, in a fishery, the environmental state could indicate the abundance and distribution of fish species. The collective impact of multiple groups (cooperators, defectors, loners) engaging in fishing activities is of greater significance for the fish population than the individual contributions of each group. The quadratic terms ($x^{2}$, $y^{2}$, $z^{2}$) denote the adverse consequences of any dominant strategy on the population, potentially resulting in excessive exploitation. The terms (xy, yz, zx) represent the positive effects of strategy diversity, which can enhance the utilization of resources in a sustainable manner. This situation succinctly describes how strategy interactions impact the environment without introducing unnecessary complexity by incorporating strategy-specific effects [27,47]. However, the case for distinct environment replenishment rates is briefly touched upon in appendix D.

The EF function $f\left(x,y,z\right)$ is essential for connecting population dynamics with environmental changes. The practical importance of this lies in its capacity to simulate intricate eco-evolutionary interactions observed in diverse natural and social systems. For instance, in microbial communities, the function refers to how various bacterial strains (similar to our strategies) influence and are influenced by their shared environment, such as the availability of nutrients or the levels of pH [24,54]. Within social contexts, this model can demonstrate how various behavioural strategies within a population can impact and adapt to evolving social norms or institutional frameworks [28,29]. The function’s structure, which incorporates both positive interactions (mixed terms) and negative self-interactions (quadratic terms), aligns with ecological models of species interactions and carrying capacity [22,23]. This formulation enables the representation of complex dynamics, such as environmental tipping points and cyclical behaviours, which have been documented in different complex systems [27,47].

By combining equation (2.6*a*) to (2.6*c*) and equation (2.8*b*), we can derive the complete system of differential equations that govern the game in equation (2.9),


(2.9*a*) $$\frac{dx}{dt}=x\left(\pi_{x}-\pi \right),$$

(2.9*b*) $$\frac{dy}{dt}=y\left(\pi_{y}-\pi \right),$$

(2.9*c*) $$\frac{dz}{dt}=z\left(\pi_{z}-\pi \right),$$

(2.9*d*) $$\frac{dn}{dt}=n\left(1-n\right)f\left(x,y,z\right),$$

where the term n(1-n) ensures n is bounded between 0 and 1.

## 3. Results and discussion

**Figure 1.**
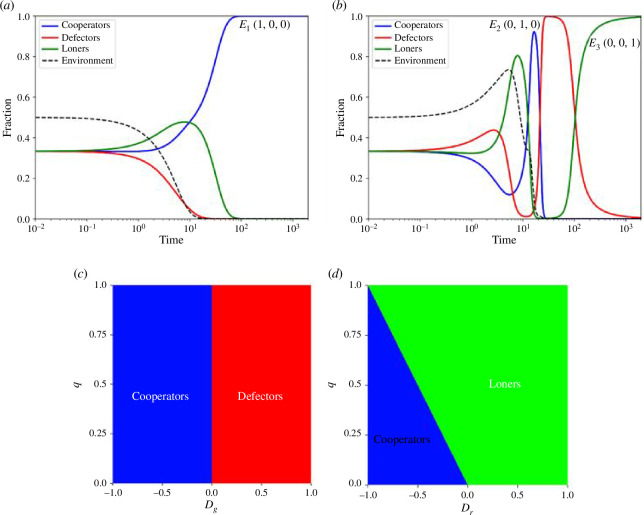
(*a*) Time-series plot showing the stable equilibrium $E_{1}\left(1,0,0\right)$. The vertical axis represents fractions of cooperators, defectors and loners in blue, red and green, respectively. The parameters are selected as $D_{g}=-0.5$, $D_{r}=0$*,* q=0.9, ϵ=0.5, θ=0.1 (*b*) Time-series plots showing the unstable equilibrium $E_{2}\left(0,1,0\right)$ and stable equilibrium $E_{3}\left(0,0,1\right)$. The vertical axis represents fractions of cooperators, defectors and loners in blue, red and green, respectively. The parameters are selected as $D_{g}=0.5$, $D_{r}=1$*,* q=0.1, ϵ=1, θ=0.9 (*c*) Highest pay-off receiving strategy in high cooperation setting (x→1) as a function of loner pay-off q and GID $D_{g}$. The region indicated in blue represents cooperation as the highest pay-off receiving strategy, whereas the region in red represents defection receiving the highest pay-off. (*d*) Highest pay-off receiving strategy in high defection setting (y→1) as a function of loner pay-off q and risk averting dilemma $D_{r}$. The region indicated by blue represents cooperation as the highest pay-off receiving strategy, whereas the region in green represents loners receiving the highest pay-off.

The stability of the game’s equilibrium is primarily determined by the environmental condition, denoted by the variable n. The game equilibriums are determined in appendix A. In a state of environmental depletion where n equals zero (n=0), the game exhibits three pure strategy Nash Equilibria. These equilibria are characterized by the dominance of cooperators $\left(E_{1}=\left(x_{1}^{*},y_{1}^{*},z_{1}^{*}\right)=\left(1,0,0\right)\right)$, defectors $\left(E_{2}=\left(x_{2}^{*},y_{2}^{*},z_{2}^{*}\right)=\left(0,1,0\right)\right)$ and loners ($E_{3}=\left(x_{3}^{*},y_{3}^{*},z_{3}^{*}\right)=(0,0,1)$). Figure 1*a* illustrates a time-series plot for a specific instance of equilibrium $E_{1}$, while figure 1*b* depicts time-series plots capturing the emergence of $E_{2}$ and $E_{3}$. The graph in figure 1*b* indicates that equilibrium $E_{2}$ is unstable, a phenomenon attributed to the differing pay-offs that strategies receive in various environmental settings.

In a scenario characterized by high cooperation $\left(x\to 1\right)$, the pay-off for a cooperator is 1, for a defector, it is $1+D_{g}$, and for a loner, it is q. For $-1\leq D_{g}\leq 1$ and 0<q<1, defectors consistently achieve the highest pay-off when $D_{g}>0$, incentivizing an increase in the population of defectors. Conversely, cooperators attain the highest pay-off when $D_{g}<0$, encouraging cooperative behaviour. In both cases, loners do not establish dominance in the scenario. Figure 1*c* visually represents this dynamic. The stability of the pure cooperation equilibrium is contingent on the value of $D_{g}$, determining whether cooperation prevails or not.

On the other hand, in a situation marked by high defection (y→1), the pay-offs for cooperators, defectors and loners are $-D_{r}$, 0 and q, respectively. The specific values of $D_{r}$ and q determine which strategy attains the highest pay-off, as depicted in figure 1*d*. Cooperators receive the maximum pay-off when $D_{r}<0$ and $q+D_{r}<0$. In all other cases, loners achieve the highest pay-off. Notably, defectors do not accept any pay-off in a highly defective setting. Consequently, the equilibrium stemming from the defection strategy is inherently unstable. This distribution of pay-offs under various high-fraction settings highlights the cyclic nature of the Prisoner’s Loner’s Game (PLG), establishing its comparability to RPS and justifying their connection through EF.

**Figure 2.**
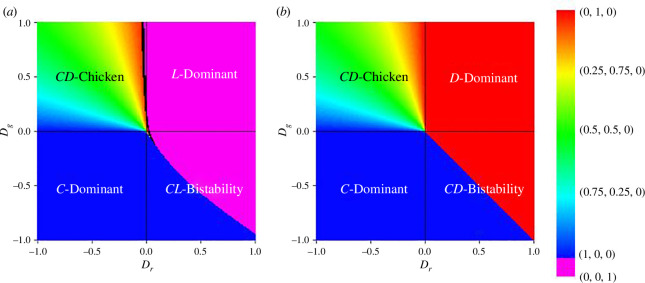
(*a*) Phase diagram of the proposed game dynamics over $D_{g}$ and $D_{r}$. Other parameters are selected as q=0.1, ϵ=0 and θ=0, (*b*) phase diagram for a two-player two-strategy game over analogous dilemma parameters. In both figures, black indicates a neutral game, purple indicates a loner-dominant scenario and the spectral transition from blue to red indicates a gradual transition from a pure cooperation setting to a pure defection setting. The inclusion of a loner strategy and EF reduces defection behaviour and replaces pure defection with a pure loner strategy.

The presence of dilemma within the game leads to the coexistence of different strategies. Figure 2*a* illustrates a phase diagram depicting the game’s dynamics about $D_{g}$ and $D_{r}$. To provide context, we include a similar depiction of game dynamics for a two-player, two-strategy game defined through a dilemma [55]. Upon comparison, we observe that both games’ portions representing pure cooperation are relatively similar when both dilemma variables are negative. When $D_{g}$ is positive but $D_{r}$ is negative, both games exhibit a coexistence reminiscent of the chicken game [55]. However, all such coexistences involve only cooperation and defection, as indicated by the phase diagrams in figure 1*c*,*d*, suggesting that loners do not emerge for negative values of $D_{r}$ and positive values of $D_{g}$ unless q is sufficiently high. This condition, however, is precisely what is required for coexistence. Figure 2*c* presents a time-series plot exemplifying one such case of coexistence. The equilibrium in this scenario is denoted by $E_{4}=\left(x_{4}^{*},y_{4}^{*},z_{4}^{*}\right)=\left(D_{r}/D_{r}-D_{g},\;-D_{g}/D_{r}-D_{g},0\right)$ where $D_{g}\neq D_{r}$.

An intriguing observation arises when considering $D_{r}>0$. In the region where $D_{g}>0$, figure 2*b* indicates pure defection resulting from a PD situation. However, the corresponding area in the phase diagram of figure 2*a* reveals a state dominated by loners. This pattern also holds for the stag hunt-type game, represented by $D_{g}<0$ and $D_{r}>0$. In figure 2*b*, most of the region marked by defection dominance transforms into an area dominated by loners in figure 2*a*. This alignment is further substantiated by the phase diagrams in figure 1*c*,*d*, demonstrating loner dominance for positive values of $D_{r}$.

**Figure 3.**
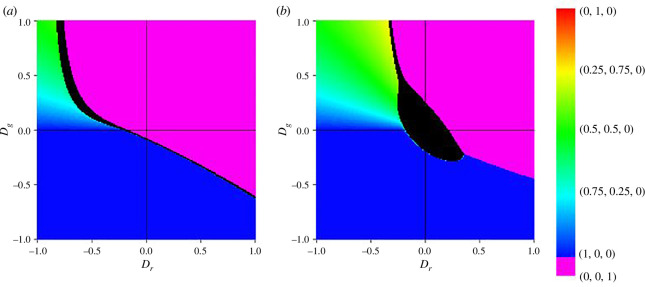
Phase diagram of the proposed game dynamics over $D_{g}$ and $D_{r}$. In both figures, black indicates a neutral game, purple indicates a loner dominant scenario and the spectral transition from blue to red indicates a gradual transition from pure cooperation to pure defection setting. (*a*) An extended region of loner dominance is observed due to the promotion of loners by a high number of defectors. Other parameters are selected as ϵ=-1, q=0 and θ=0. (*b*) Black region denoting neutral game where all strategies receive an equal pay-off. Other parameters are selected as ϵ=-1, q=0.1 and θ=0.9.


Figure 3 presents another phase diagram resembling figure 2*a* but with different parameter combinations. Figure 3*a* illustrates an expanded loner-dominant region encompassing a significant portion of the chicken game. Our earlier demonstration established that a defector-dominant environment can quickly be overtaken by loners, whereas a cooperation-dominant environment cannot. This explains why the coexistence of defectors strongly promotes the prevalence of loners. In various phase diagrams, a distinctive black region emerges, characterized by the equilibrium $E_{5}=\left(x_{5}^{*},y_{5}^{*},z_{5}^{*}\right)=\left(1/3,1/3,1/3\right)$, representing equal fitnesses for all strategies. In specific situations, such as when the initial population is $\left(x_{0},y_{0},z_{0}\right)=\left(1/3,1/3,1/3\right)$ and $D_{g}=D_{r}=0$ (centre of the phase diagram in figure 3*b*), there is no change in the fraction of strategies according to mean-field approximation. In such cases, cooperators and defectors receive $\pi_{x}=\pi_{y}=\frac{1}{3}+\frac{1}{3}q$, while loners receive q. When q takes specific values, for example q=0.5, all strategies’ fitnesses become equal. The precise value of q for which this phenomenon occurs is determined by the conditions in equation (3.1*a*), which is derived in appendix C. This type of game is considered neutral, where the population tends to adhere to its respective choices.

(3.1*a*) $$q=\frac{D_{r}y-x}{z-1}=\frac{-x\left(D_{g}+1\right)}{z-1},$$

(3.1*b*) $$xD_{g}+yD_{r}=0.$$

**Figure 4.**
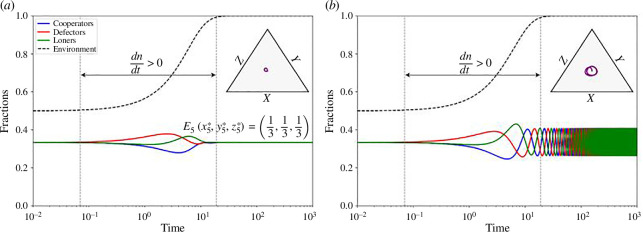
(*a*) Time-series plot showing the equal fraction equilibrium or the no dominance equilibrium $E_{5}\left(1/3,1/3,1/3\right)$ in a replenished environment (n=1). Other parameters are $D_{g}=-1$, $D_{r}=-1$, ϵ=-1, q=0.5 and θ=0.9. (*b*) Time-series plots showing sustained oscillations that satisfy equation (3.2) in a replenished environment (n=1) with ϵ=0. Other parameters are $D_{g}=1$, $D_{r}=1$, q=0.9 and θ=0.9. In both figures, the vertical axis represents fractions of cooperators, defectors and loners in blue, red and green, respectively.

Under a replenished environment where n=1, the game exhibits rock paper scissor cyclic dominance. In this case, we find the equilibrium $E_{5}=\left(x_{5}^{*},y_{5}^{*},z_{5}^{*}\right)=\left(1/3,1/3,1/3\right)$ as the stable equilibrium. Illustrated in figure 4*a*, the population exhibits behaviour characteristic of a neutral game. This behaviour persists as long as n remains in an equilibrium state. However, when the value of n changes, the population fraction experiences a transient shift before ultimately returning to equilibrium. Notably, this equilibrium is predominantly observed for ϵ<0, a point that will be elaborated on later in the discussion.

Another observed scenario is the cyclic oscillation in a replenished environment, depicted in figure 4*b*. This phenomenon occurs when ϵ=0 and is governed by the solution to the equation presented in (3.2):


(3.2) $$\theta \left(2xy+2yz+2zx\right)=x^{2}+y^{2}+z^{2}.$$

Equation (3.2) is very important in the context of the game dynamics. Because of how the environmental influence function is defined, the environment is replenished (dn/dt>0) when the population fractions are closer to each other. As soon as one fraction of the population significantly increases or decreases compared to others, f<0, which leads to dn/dt<0, causing the environment to deplete. Some important details about the environmental influence function are touched upon in appendix B.

**Figure 5.**
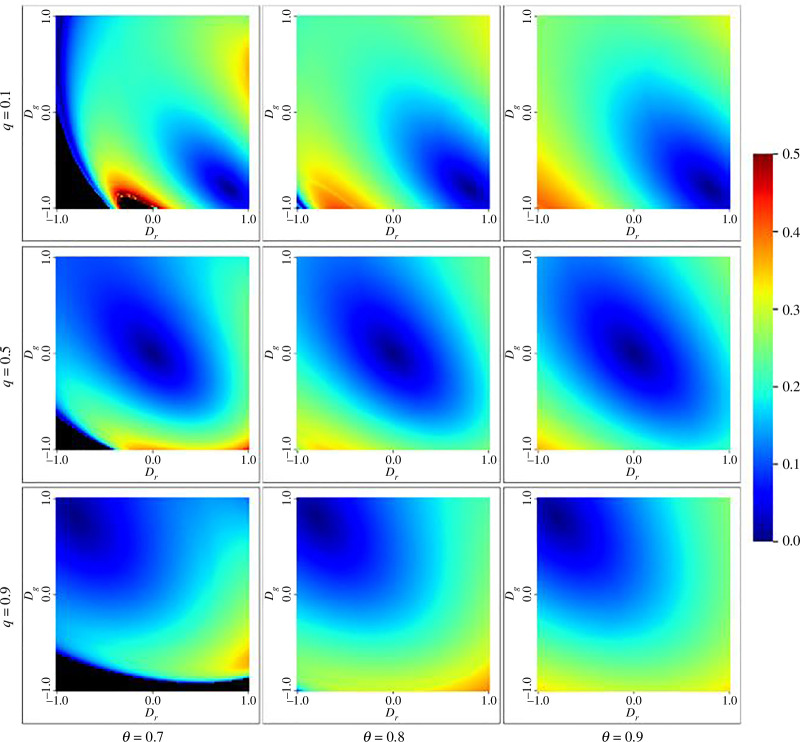
Heatmap showing the amplitude of oscillations in the replenished environment (n=1) as a function of $D_{g}$ and $D_{r}$ with varying q, ϵ=0, and θ→1. Black region represents depleted environment without oscillation.

The amplitude of the oscillating scenario is characterized by θ, q, $D_{g}$ and $D_{r}$. In figure 5, we present the plot of amplitudes as the functions of the parameter. The oscillation takes place for θ→1. We can observe from figure 5 that the oscillation tends to be minimal around points where $D_{g}=D_{r}$. As the value of q fluctuates, there is a lateral translation of the phase space along the straight line $D_{g}-D_{r}=0$.

**Figure 6.**
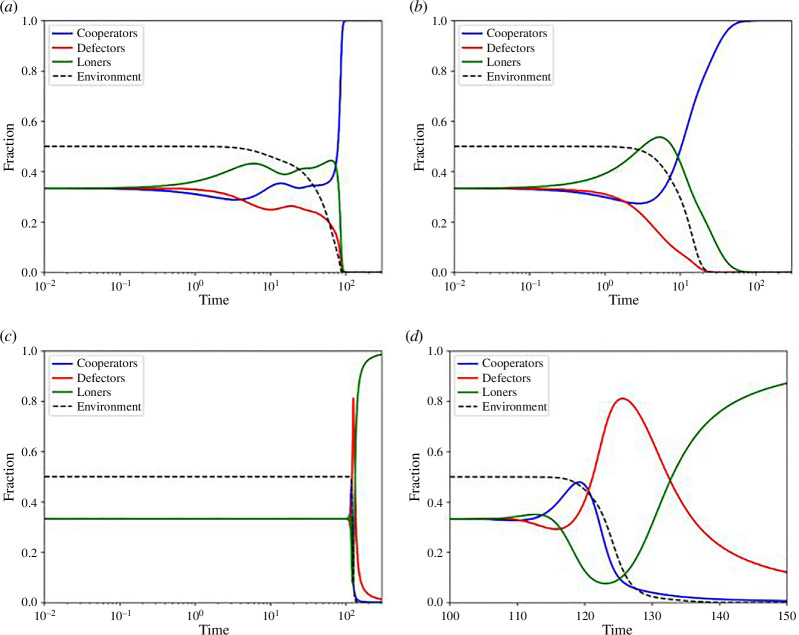
(*a*) Time-series plot showing rapid environmental depletion hindering the formation of equilibrium $E_{6}$ for ϵ≠0. Other parameters are $D_{g}=-0.5$, $D_{r}=1$, q=0.5 and θ=0.5. (*b*) Time-series plot showing rapid environmental depletion hindering the formation of equilibrium $E_{6}$ for ϵ=0. Other parameters are $D_{g}=-0.5$, $D_{r}=1$, q=0.9 and θ=0.5. (*c*) Time-series plot demonstrating rapid declination of environment. Other parameters are $D_{g}=D_{r}=0$, q=0.5, ϵ=2 and θ=0.5. The game dynamics appear as a neutral game until minor fluctuations in population fractions cause chaotic changes in the overall dynamics. (*d*) Zoomed view highlighting chaotic fluctuations in figure 6*c* from t=100 to t=150. In both figures, the vertical axis represents fractions of cooperators, defectors and loners in blue, red and green, respectively.

In the context of a replenished environment, a variant of the chicken game seeks to emerge, characterized by the parameter ϵ. The theoretically derived equilibrium, denoted as $E_{6}=\left(x_{6}^{*},y_{6}^{*},z_{6}^{*}\right)=\left(\epsilon -1/2\epsilon ,0,\epsilon +1/2\epsilon \right)$, arises when conditions such as $\pi_{x}=\pi_{z}=\pi$, y=0 and ϵ≠0 are met in the RPS game. The cyclic nature of the game theoretically implies the existence of two other variants of this equilibrium based on fitness and pay-offs, namely (0,ϵ+1/2ϵ,ϵ−1/2ϵ) and (ϵ+1/2ϵ,ϵ−1/2ϵ,0). However, none of these equilibria are observed. For the coexistence of multiple strategies, either n=0 or ϵ=0 needs to be fulfilled, both of which are violated in this scenario. Moreover, the equilibrium $E_{6}$ exhibits a significantly higher population fraction (either cooperators or loners) than the others. Consequently, when the $E_{6}$ equilibrium attempts to form in a replenished state, the environment depletes, negating the possibility of $E_{6}$ formation. In figure 6, a time-series plot illustrates this phenomenon. Figure 6*a* depicts a case where ϵ≠0, and figure 6*b* showcases a chance with ϵ=0. In both instances, the environment begins to recede as soon as a particular fraction becomes excessively high. In the case of ϵ≠0, cyclic oscillations are observed before the environment ultimately depletes. Figure 6*c*,*d* further illustrate the rapid depletion of the domain. Figure 6*c* presents the overall graph, while 6*d* zooms in on the period from t=100 to t=150. In contrast to previous instances, the game appears stable with the $E_{5}$ equilibrium until a slight adjustment disrupts the equilibrium, causing rapid environmental depletion and the end of the neutral game.

**Figure 7.**
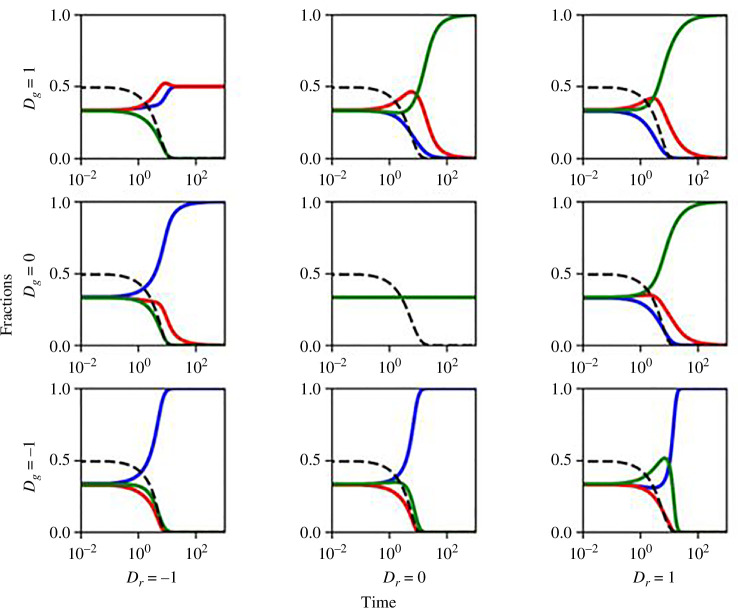
Time-series plots of the game dynamics and outcomes for depleted environment scenario $\left(n=0\right)$ with θ<0.5. The vertical axis represents fractions of cooperators, defectors and loners in blue, red and green, respectively. $D_{r}$ is changed along the plots horizontally and $D_{g}$ vertically. The environment is depleted due to θ being low, and the game dynamics and outcomes are determined by $D_{g}$, and $D_{r}$. Negative values of $D_{g}$ promote $E_{1}\left(1,0,0\right)$ equilibrium. For positive values of $D_{g}$, negative $D_{r}$ promotes mixed strategy equilibrium $E_{4}\left(D_{r}/D_{r}-D_{g},-D_{g}/D_{r}-D_{g},0\right)$, whereas positive $D_{g}$ promotes loner dominant equilibrium $E_{3}\left(0,0,1\right)$. $D_{g}=D_{r}=0$ leads to $E_{5}\left(1,3,1,3,1,3\right)$. The other parameters are selected as θ=0.1 and q=0.5.

Now, our analysis strongly indicates that the equilibrium and endgame outcomes in the PLG with EF hinge significantly on the state of the environment. The state of the environment is governed by two key parameters, θ and ϵ. When θ<0.5, environmental depletion is assured, whereas θ approaching 1 guarantees environmental replenishment. Our subsequent discussions revolve around the overarching influence of these parameters on the game dynamics. Figure 7 illustrates the scenario for θ<0.5. In this case, the game dynamics are primarily determined by the values of $D_{g}$ and $D_{r}$. Depending on the dilemma values, the game can exhibit characteristics of the chicken game, cooperator-loner stag hunt, a trivial solution or loner dominance. Notably, the situation where a two-strategy game becomes a PD with defector dominance is no longer applicable in the three-strategy game. This highlights a significant advantage of incorporating the loner strategy—it ensures the existence of cooperation and the extinction of defects, as high defectors allow for a higher fitness for loners even with a relatively small q. Another important insight from figure 7 is that the environment is still depleted even when a neutral game is observed. A neutral game occurs in figure 7 when $D_{g}=D_{r}=0$, satisfying the conditions in equation (3.1). However, with θ being too low, environmental depletion occurs irrespective of how the population is distributed.

**Figure 8.**
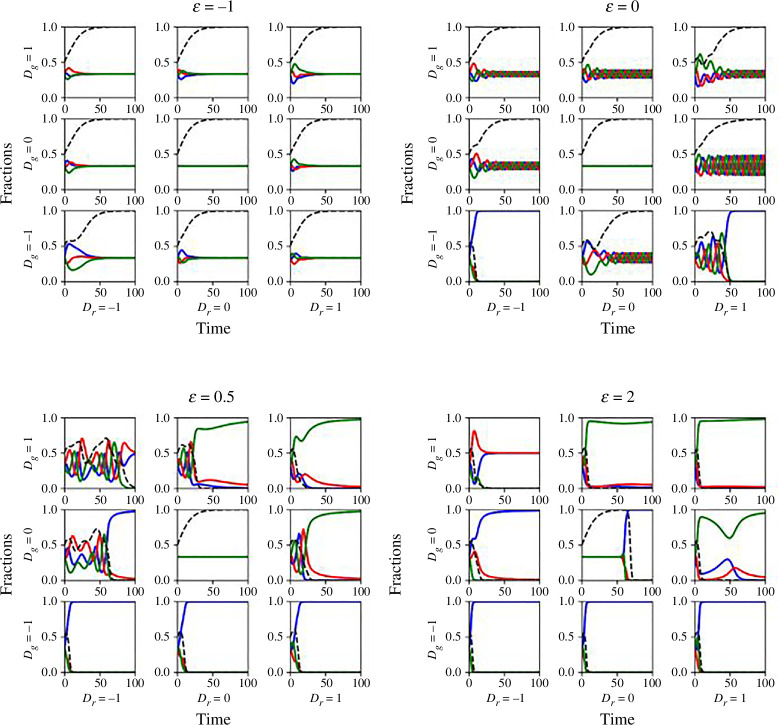
Time-series plots for game dynamics for θ>0.5 but not considering θ→1. The vertical axis represents fractions of cooperators, defectors and loners in blue, red and green, respectively. $D_{r}$ is changed along the plots horizontally and $D_{g}$ vertically. The value of ϵ dictates the game. ϵ<0 promotes draw games over confrontation games, and the ultimate game dynamic is $E_{5}\left(1/3,1/3,1/3\right)$ in a replenished environment (n=1). ϵ=0 either depletes the environment, leading to a dilemma-dictated outcome or promotes cyclic oscillations in a replenished environment. 0<ϵ<1 promotes chaos with fluctuating population strategies and fluctuating environmental variable n before the environment eventually depletes, and dilemma shapes the game outcome. ϵ>2 promotes confrontation game over draw game, depleting the environment and resulting in outcome determined by $D_{g}$ and $D_{r}$.

In contrast to the previous scenario, θ→1 consistently guarantees environment replenishment, regardless of population distribution. Our previous discussions and results for such environments are detailed in figures 4 and 5. However, when θ is midway between 0.5 and 1, intriguing outcomes arise, strongly influenced by the parameter ϵ. Figure 8 presents four different cases in this scenario. The primary role of ϵ is to determine the strategies players would prefer in an environment-replenished RPS game. When ϵ<0, a draw game yields the same pay-off as losing in a confrontation game. In this case, population fractions initially change due to different pay-offs for different strategies, while dn/dt>0, but the fractions eventually settle to the $E_{5}=\left(x_{5}^{*},y_{5}^{*},z_{5}^{*}\right)=\left(1/3,1/3,1/3\right)$ equilibrium as soon as n=1 is achieved. After reaching this equilibrium, any individual has no incentive to change strategies given the pay-offs and environmental conditions. Everyone receives a pay-off of $\pi_{x}=\pi_{y}=\pi_{z}=\epsilon /3$, resulting in π=ϵ/3 and dx/dt=dy/dt=dz/dt=0.

When ϵ=0, the game is a pure RPS game and should have behaved like a neutral game if the initial population is $\left(x_{0},y_{0},z_{0}\right)=\left(1/3,1/3,1/3\right)$. However, the observed dynamics deviate due to EF and the dilemma, showcasing cyclic dominance with oscillations. As previously discussed, the dilemma variables also influence the amplitude of this oscillation. This amplitude is contingent on the fractions of each strategy at the beginning of the pure RPS situation, determined by the values of $D_{g}$ and $D_{r}$ during the dn/dt>0 phase. Consequently, $D_{g}<0$ can result in a significant promotion of cooperators and the eventual depletion of the environment. In the case where 0<ϵ<1, the game’s states are primarily determined by the dn/dt≠0 phase. Within low ranges of $D_{r}$ and $D_{g}$, attempts to form a chicken game are hindered by environmental constraints, leading to high-frequency fluctuations among strategy frequencies. These fluctuations, chaotic in nature, persist until the environment depletes (n=0). The values of $D_{g}$and $D_{r}$ then determine the game’s final outcome. In most cases in ϵ∈(0,1), chaotic cyclic dominance prevails unless $D_{g}$, $D_{r}$ and q allow cooperation or loners to dominate the game. The chaotic cyclic power is associated with the equilibrium $E_{6}$, which has been discussed as impossible to form. When ϵ>1, a draw game is considered better than winning in a confrontation game. This leads the population to engage more frequently in draw games, causing the population fractions to deviate further from the no-dominance equilibrium $E_{5}$. Consequently, the environment rapidly depletes, and the values of $D_{g}$ and $D_{r}$ soon determine the game’s outcome.

## 4. Conclusion

The study elucidates an intriguing expansion of the classic PD into a three-strategy game by including a loner strategy coupled with universal dilemma scaling and EF. Our results demonstrate that cyclic dominance readily materializes in this framework, fundamentally shifting evolutionary outcomes to promote cooperation over defection. The PD game provides a paradigm for studying conflicts between cooperation and defection in biology and psychology [5,56–60]. Without a third strategy [6–8], a ‘tit-for-tat’ mechanism [4,5,61], or reciprocity mechanisms [62], defection outcompetes cooperation. However, introducing a third strategy often induces cyclic dominance, resembling RPS games. RPS games inherently have the nature of OPD built into them. For example, Kirkup demonstrated RPS interactions between antibiotic-producing, antibiotic-sensitive and antibiotic-resistant *Escherichia coli* strains in mouse intestines [54]. Antibiotic-producing bacteria overpower antibiotic-sensitive bacteria. Antibiotic-resistant organisms proliferate and endure, outperforming antibiotic-producing microorganisms, which allows antibiotic-sensitives to multiply and surpass others. This cycle continues until the antibiotic-producing organisms reproduce once more. This cyclic competition resembles an OPD: defectors harming cooperators, loners immune to defection and cooperators outperforming loners. However, there are not many studies merging OPD and RPS games, let alone using EF. Per Su *et al*., distinct games can be hybridized, motivating our framework by combining OPD and RPS through EF [40].

Our results highlight that the dilemma parameters $D_{g}$ and $D_{r}$ primarily dictate game dynamics in a depleted environment, leading to outcomes resembling the Chicken game, Stag Hunt or loner dominance. However, EF enables oscillations, coexistence and chaos as the environment shifts towards a replenished state. The feedback parameters play a key role—controlling the environment’s tendency to replenish or deplete while adjusting pay-offs for draw games. Specific configurations of the core dilemma strengths and environmental parameters provoke intriguing dynamical phenomena. Rapid, chaotic fluctuations, sustained oscillations and balanced coexistence can emerge under certain settings as the environment shifts from depleted to replenished. However, defectors consistently experience extinction. This contrasts sharply with traditional two-strategy social dilemmas where defection persists. Specific parameter configurations lead to intriguing phenomena, including rapid, chaotic fluctuations, cyclic oscillations and neutral ‘balancing acts.’ This demonstrates that the option to abstain coupled with environmental constraints significantly alters evolutionary outcomes compared with traditional two-strategy social dilemmas. Defection is no longer evolutionarily favourable. Instead, cyclic dominance ensues, promoting cooperation. Our framework and results have implications for studying adaptation and decision-making in contexts involving environmental pressures. The transitions in dynamics could model phenomena across domains like economics, ecology and sociology. Further investigations could focus on specific case studies and applications to validate the utility of this evolutionary game theory approach augmented by EF.

The dynamics transitions observed in our model exhibit significant similarities with natural phenomena in various domains. The phenomenon of cyclic dominance in economics can represent the fluctuation of various business strategies in a competitive market with limited resources. Companies may employ different strategies in the technology sector depending on market conditions. These strategies include innovation (cooperation), imitation (defection) and diversification (loner strategy) [31,63]. Our ecological model exhibits dynamics that mirror the cyclical patterns observed in species populations involved in predator–prey relationships or competitive ecosystems [22,23,64]. The EF in our model is similar to how species interactions can modify their shared habitat, thereby influencing future population dynamics. Within the field of sociology, the model serves as a representation of the progression of social norms or behaviours in reaction to shifting societal circumstances. During periods of resource scarcity, individualistic behaviours tend to prevail, whereas, in times of abundance, more cooperative or diverse behaviours are likely to arise [29,30,65].

Although our model offers valuable insights into these intricate systems, it is crucial to recognize its limitations. The interactions between environmental factors in the real world are frequently more complicated than our simplified depiction. Factors such as variations in space, delays in time and random effects, which are not accounted for in our current model, can significantly impact the system’s dynamics [21,59]. Furthermore, the assumption that a population is well-mixed may not be valid in numerous real-world situations where local interactions are paramount.

Although there are some limitations, our work establishes a basis for future research on the interaction between strategic behaviour and EF. Subsequent research endeavours have the potential to expand upon this model by incorporating spatial organization, diverse individuals or intricate ecological patterns. Conducting empirical validation using case studies in specific domains is essential for improving the model and increasing its predictive accuracy. This line of research has the potential to significantly enhance our understanding and potentially enable us to effectively manage complex adaptive systems in nature and society by connecting theoretical models with real-world phenomena.

The transitions in dynamics have salient parallels with natural phenomena across diverse domains like economics, ecology and sociology. The proposed game-theoretic approach, therefore, holds significant promise for gaining fundamental insights into adaptation, decision-making and systemic behaviours in contexts involving environmental pressures and constraints. Further research should focus on validating the model through case studies and real-world applications. Fine-tuning dilemma parameters and environmental variables to match empirical observations will be essential. Investigations could also consider increasingly complex versions, for example by incorporating spatial structure or heterogeneous agents. Such extensions may reveal additional mechanisms and outcomes beyond cyclic dominance that facilitate the evolution of cooperation [21,61]. This study puts forth an essential step towards more realistically capturing the interplay between evolutionary game dynamics and EF. Our findings highlight that the option to abstain can profoundly influence collective outcomes, with valuable implications across the natural and social sciences.

## Figures and Tables

**Figure 9.**
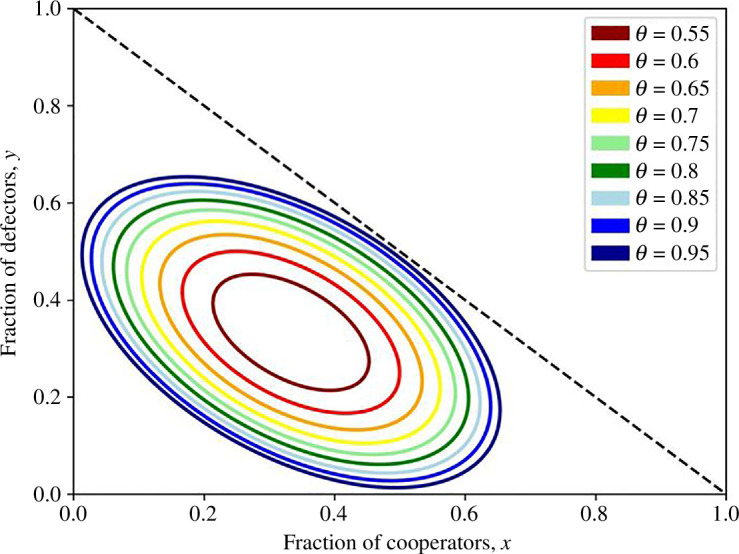
Family of curves generated by equation (B1) of the electronic supplementary material. The environmental replenishment rate θ parametrizes the curves. For any curve generated by θ, the area bound by the curve represents population fractions replenishing the environment, whereas the area outside the curve represents the depletion of the environment.

**Figure 10.**
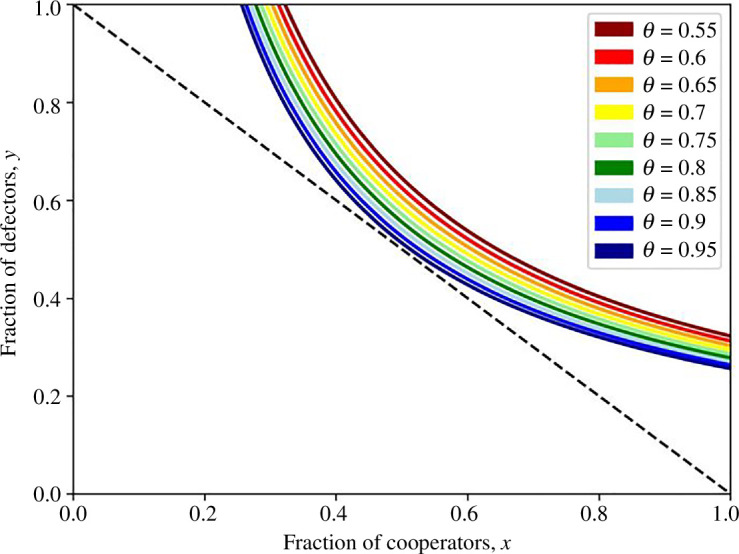
Family of curves generated by equation B2 of the electronic supplementary material. Since none of the curves lie within the x+y≤1 region, no game dynamic is observable from the equation.

**Figure 11.**
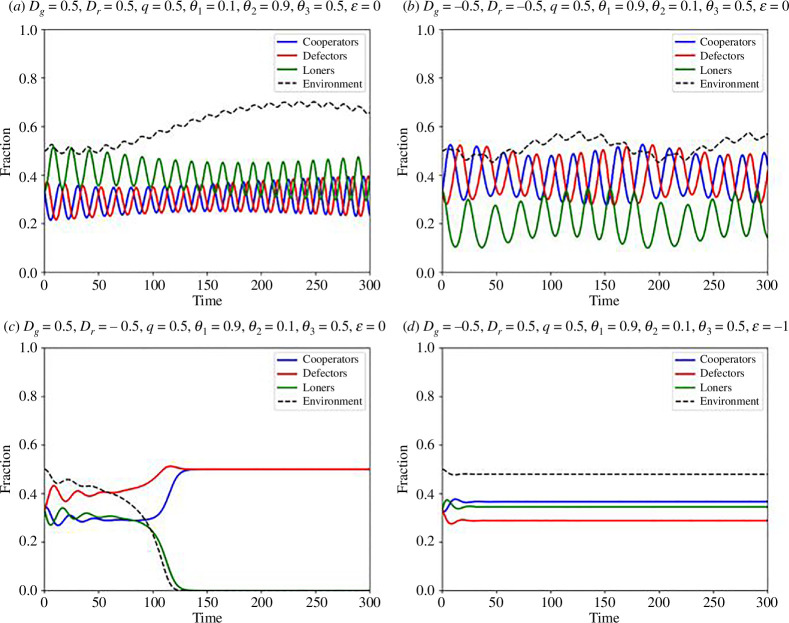
Game dynamics for distinct environmental rates. Figure (*a*) and (*b*) represents two different cases of oscillating game dynamics where the environment oscillates as well. Figure (*c*) shows an attempt to form a mixed strategy equilibrium in replenished environment which does not formulate due to environmental tipping point. However, under the right parameter combination, as seen in figure (*d*), the mixed equilibrium sustains with partial environmental resources.

## Data Availability

The datas are available online [66].

## Appendix A. Equilibrium conditions of the pairwise game

The environment-dependent pay-off matrix of the game is selected as:

(A 1)
$$A\left(n\right)=\left(1-n\right)A_{PLG}+nA_{RPS},$$

where $A_{PLG}$ is the pay-off matrix of a PLG developed from OPD rescaled with universal dilemma scaling, and $A_{RPS}$ is the pay-off matrix of an RPS game with modified draw game pay-off. The matrices are as follows:

(A 2*a*)
$$A_{PLG}=\left[\begin{array}{ccc} 1 & -D_{r} & q\\ 1+D_{g} & 0 & q\\ q & q & q \end{array}\right],$$

(A 2*b*)
$$A_{RPS}=\left[\begin{array}{ccc} \epsilon & -1 & 1\\ 1 & \epsilon & -1\\ -1 & 1 & \epsilon \end{array}\right].$$

$D_{g}$ is the GID variable, $D_{r}$ is the risk averting dilemma, q is the loner pay-off and ϵ is the draw game pay-off. Putting the values from equation (A 2) in equation (A 1), the pay-off matrix can be rewritten as:

(A 3)
$$A\left(n\right)=\left[\begin{array}{ccc} \left(1-n\right)+n\epsilon & -\left(1-n\right)D_{r}-n & \left(1-n\right)q+n\\ \left(1-n\right)\left(1+D_{g}\right)+n & n\epsilon & \left(1-n\right)q-n\\ \left(1-n\right)q-n & \left(1-n\right)q+n & \left(1-n\right)q+n\epsilon \end{array}\right].$$

Considering x, y and z as the fractions of cooperators (rock), defectors (paper) and loners (scissors), respectively, the fitnesses are:

(A 4*a*)
$$\pi_{x}=\left[\left(1-n\right)+n\epsilon \right]x+\left[-\left(1-n\right)D_{r}-n\right]y+\left[\left(1-n\right)q+n\right]z,$$

(A 4*b*)
$$\pi_{y}=\left[\left(1-n\right)\left(1+D_{g}\right)+n\right]x+n\epsilon y+\left[\left(1-n\right)q-n\right]z,$$

(A 4*c*)
$$\pi_{z}=\left[\left(1-n\right)q-n\right]x+\left[\left(1-n\right)q+n\right]y+\left[\left(1-n\right)q+n\epsilon \right]z,$$

(A 4*d*)
$$\pi =x\pi_{x}+y\pi_{y}+z\pi_{z}.$$

The environmental influence function stands as:

(A 5)
$$f\left(x,y,z\right)=\theta \left(2xy+2yz+2zx\right)-\left(x^{2}+y^{2}+z^{2}\right),$$

where θ is the environment replenishment rate. The population and environment dynamics are given as:

(A 5*a*)
$$\frac{dx}{dt}=x\left(\pi_{x}-\pi \right),$$

(A 5*b*)
$$\frac{dy}{dt}=y\left(\pi_{y}-\pi \right),$$

(A 5*c*)
$$\frac{dz}{dt}=z\left(\pi_{z}-\pi \right),$$

(A 5*d*)
$$\frac{dn}{dt}=n\left(1-n\right)f\left(x,y,z;\theta \right).$$

We set equation (A 5*d*) to zero to find the equilibrium points. It follows:

(A 6)
n(1−n)f(x,y,z;θ)=0.

We get three cases from here.

### **Case 1:** n=0

The pay-off matrix is identical to an OPD game with universal dilemma scaling. Using x+y+z=1, the population fitness functions reduce to:

(A 7*a*)
$$\pi_{x}=x-D_{r}y+q\left(1-x-y\right)=\left(1-q\right)x+\left(-D_{r}-q\right)y+q,$$

(A 7*b*)
$$\pi_{y}=\left(1+D_{g}\right)x+q\left(1-x-y\right)=\left(1+D_{g}-q\right)x-qy+q,$$

(A 7*c*)
$$\pi_{z}=q.$$

The replicator equations are:

(A 8*a*)
$$\frac{dx}{dt}=x\left(\pi_{x}-\pi \right),$$

(A 8*b*)
$$\frac{dy}{dt}=y\left(\pi_{y}-\pi \right).$$

We set equations (2.8*a*) and (2.8*b*) to zeroes for stable solution. This gives three pure strategy equilibrium conditions: all cooperators $E_{1}=\left(x_{1}^{*},y_{1}^{*},z_{1}^{*}\right)=\left(1,0,0\right),$ all defectors $E_{2}=\left(x_{2}^{*},y_{2}^{*},z_{2}^{*}\right)=\left(0,1,0\right)$ and all loners $E_{3}=\left(x_{3}^{*},y_{3}^{*},z_{3}^{*}\right)=\left(0,0,1\right)$.

Furthermore, setting $\pi_{x}=\pi_{y}=\pi$ and z=0 to satisfy equations (2.8*a*) and (2.8*b*), we get the mixed strategy equilibrium $E_{4}=\left(x_{4}^{*},y_{4}^{*},z_{4}^{*}\right)=\left(D_{r}/-D_{g}+D_{r},-D_{g}/-D_{g}+D_{r},0\right)$.

### **Case 2:** n=1

The pay-off matrix is identical to an RPS game with a non-zero draw pay-off. Consider x=y=0 and $\pi_{z}=\pi$. These conditions satisfy dx/dt=dy/dt=dz/dt=0 from equations (2.6a), (2.6b) and (2,6c). Solving these conditions gives the equilibrium $E_{3}=\left(0,0,1\right)$. The other pure strategy equilibriums are obtainable in this manner by cyclically changing x, y and z. However, because we assume high population fractions deplete the environment, no pure strategy equilibria can be observed under replenished conditions.

Equating $\pi_{x}=\pi_{y}=\pi_{z}=\pi$, we get,

(A 9)
(ϵ−1)−2y+1=2x+(ϵ+1)y−1=−(ϵ+1)x+(−ϵ+1)y+ϵ.

Solving equation (A 9) for x and y provides the mixed strategy equilibrium $E_{5}=\left(x_{5}^{*},y_{5}^{*},z_{5}^{*}\right)=\left(1/3,1/3,1/3\right)$ identical to the Nash equilibrium of a traditional RPS game. This equilibrium requires $\epsilon^{2}+3\neq 0$. Since ϵ∈R in this study, the equilibrium $E_{5}$ is graphically observed in our study.

Consider $\pi_{x}=\pi_{y}=\pi$ and z=0. Therefore, x+y=1, and the fitness for cooperators and defectors become as follows:

(A 10*a*)
$$\pi_{x}=\epsilon x-y=\left(\epsilon +1\right)x-1,$$

(A 10*b*)
$$\pi_{y}=x+\epsilon y=\left(1-\epsilon \right)x+\epsilon .$$

Setting equations (3.1*a*) and (3.1*b*) equal to each other and solving for x, we get the equilibrium point $E_{6}=\left(x_{6}^{*},y_{6}^{*},z_{6}^{*}\right)=\left(\epsilon +1/2\epsilon ,\epsilon -1/2\epsilon ,0\right)$. Cyclically changing x, y, and z gives us two more variations of the same scenario, namely (0,ϵ+1/2ϵ,ϵ−1/2ϵ) and (ϵ−1/2ϵ,0,ϵ+1/2ϵ). All these equilibriums require ϵ≠0. In our study, the coexistence of strategies is ensured by either (a) ϵ=0 and n=1 or (b) n=0, $D_{g}>0$ and $D_{r}<0$. Both of these conditions are in contradiction with the equilibrium $E_{6}$. Therefore, this equilibrium is not graphically observed in our study.

### **Case 3:** f=0

This case does not produce any equilibrium. The details of the environmental influence function are provided in appendix B.

## Appendix B. Environment influence function

Setting equation (A 5) equal to zero and substituting z=1-x-y gives the following equation:

(B 1)
$$-2\theta \left(x^{2}+xy+y^{2}-x-y\right)=2x^{2}+2xy+2y^{2}-2x-2y+1.$$

Equation (B 1) represents a family of ellipses parametrized by θ and centred at (1/3,1/3), shown in figure 9. The area bounded by the ellipses represents the population fraction for which the environment gets replenished, and the area outside the ellipses indicates depletion of the environment. This agrees with our assumption that a high population fraction of any one type depletes the environment. When θ<0.5, no ellipse is formed, and the environment is guaranteed to deplete. As θ→1, the region bounded by the ellipse increases, and thus, the chances of the environment being replenished increase as well.

Let us consider the case of one strategy being extinct. Without loss of generality, we assume loners will be absent from the game. This means x+y=1, and equation (B 1) becomes:

(B 2)
$$2\theta xy=x^{2}+y^{2}.$$

The family of curves denoted by equation (B 2) has no solution where x+y≤1 and 0<θ<1, although the curve tangentially approaches x+y=1 at (1/2,1/2) when θ→1. As such, no equilibrium arises from $f\left(x,y,z;\theta \right)=0$ if a strategy becomes extinct. The graphical representation is provided in electronic supplementary material, figure 10.

## Appendix C. Neutral game

Figures 2*a* and 3*a,b* have a prominent neutral game region denoted by black. We define a neutral game as the scenario when the fitnesses for all three strategies are equal, i.e. $\pi_{x}=\pi_{y}=\pi_{z}$, and therefore, there is no incentive to switch strategies. If the individual fitness becomes equal to the population fitness, the game arrives at the $E_{5}\left(1/3,1/3,1/3\right)$ equilibrium.

Under a depleted environment (n=0), the loner always receives a pay-off of q. As such, the fitness of cooperators and defectors determines the condition of a neutral game. Using z=1-x-y, the fitness of cooperators and defectors becomes as follows:

(C 1*a*)
$$\pi_{x}=x-D_{r}y+q\left(1-x-y\right),$$

(C 1*b*)
$$\pi_{y}=\left(1+D_{g}\right)x+q\left(1-x-y\right).$$

For $\pi_{x}=\pi_{y}$, we need $x-D_{r}y=\left(1+D_{g}\right)x$, which gives the following expression as the condition for a neutral game:

(C 2)
$$xD_{g}+yD_{r}=0.$$

Again, setting $\pi_{x}=q$, we get:

(C 3)
$$x-D_{r}y+qz=q.$$

Solving for q, we get the precise value of q needed for a neutral game to take place:

(C 4)
$$q=\frac{D_{r}y-x}{z-1}=\frac{-x\left(D_{g}+1\right)}{z-1}.$$

Equations (C 2) and (C 4) must be fulfilled for the neutral game to occur. Once the instantaneous values of the population fraction satisfy these conditions at any time, the rest of the simulation results in a neutral game. Considering the bound on the value of $q\in \left(0,1\right)$, equation (C 4) gives the following expressions:

(C 5*a*)
$$0<x-D_{r}y+z<1\Rightarrow 0<\pi_{x,OPD}<1,$$

(C 5*b*)
$$0<\left(1+D_{g}\right)x+z<1\Rightarrow 0<\pi_{y,OPD}<1,$$

where $\pi_{x,OPD}$ and $\pi_{y,OPD}$ are the cooperator and defector pay-offs in OPD setting, respectively.

Under a replenished environment (n=1), the neutral game occurs for most of this study’s simulations. The conditions for the neutral game in a replenished environment are the same as those for $E_{5}$ equilibrium, as discussed before.

## Appendix D. Distinct environmental replenishment rates

The environmental influence function in equation (2.8*b*) assumes uniform environment replenishment and depletion rates for all the strategies. However, a more general case would consider environment replenishment rates $\theta_{c,r}$, $\theta_{d,r}$ and $\theta_{l,r}$ for cooperators, defectors and loners, respectively. Similarly, the environment depletion rates are $\theta_{c,d}$, $\theta_{d,d}$ and $\theta_{l,d}$, respectively. Therefore, the environment influence function becomes:

(D 1)
$$f\left(x,y,z\right)=2\left(\theta_{c,r}\theta_{d,r}xy+\theta_{d,r}\theta_{l,r}yz+\theta_{l,r}\theta_{c,r}zx\right)-\left(\theta_{c,d}^{2}x^{2}+\theta_{d,d}^{2}y^{2}+\theta_{l,d}^{2}z^{2}\right)$$

Considering relative replenishment rates $\theta_{c}=\theta_{c,r}/\theta_{c,d}$, $\theta_{d}=\theta_{d,r}/\theta_{d,d}$ and $\theta_{l}=\theta_{l,r}/\theta_{l,d}$ for cooperators, defectors and loners respectively, and $\theta_{c,d}=\theta_{d,d}=\theta_{l,d}=1$ as the absolute maximum environment replenishment rates, the environmental influence functions takes the following form.

(D 2)
$$f\left(x,y,z\right)=2\left(\theta_{c}\theta_{d}xy+\theta_{d}\theta_{l}yz+\theta_{l}\theta_{c}yz\right)-\left(x^{2}+y^{2}+z^{2}\right).$$

Furthermore, substituting $\theta_{1}=\theta_{c}\theta_{d}$, $\theta_{2}=\theta_{d}\theta_{l}$ and $\theta_{3}=\theta_{l}\theta_{c}$ as the joint replenishment environmental replenishment rates, it follows:

(D 3)
$$f\left(x,y,z\right)=2\left(\theta_{1}xy+\theta_{2}yz+\theta_{3}yz\right)-\left(x^{2}+y^{2}+z^{2}\right).$$

Equation (D 3) evidently leads to different environmental tipping points compared with equation (2.8*b*). While going over all the game dynamics is beyond the scope of this study, certain results not observed in the study are presented in figure 11.
